# An Orphan Needing a Champion–Barriers to Evaluations of Decision-Making Capacity

**DOI:** 10.1093/geroni/igaa057.147

**Published:** 2020-12-16

**Authors:** Ronan Factora, Saket Saxena

**Affiliations:** Cleveland Clinic, Cleveland, Ohio, United States

## Abstract

Impaired decision making capacity is common in medical and community settings, often coexistent with functional impairment and dementia. Though the legal system determines competence, deciding if guardianship is appropriate often relies on a medical assessment of decision making capacity. Physicians conduct cognitive evaluations but are often unaware of a person’s functional impairments and are unfamiliar with their role in pursuing guardianship. Despite the individual roles of the legal system, medical system, and community agencies (e.g. Adult Protective Services (APS)) in such cases, no agency claims responsibility for ensuring this process’s fidelity. As numbers of older citizens in the US increase along with associated increasing numbers of cases involving impaired decision making capacity, ensuring this processes’ fidelity is essential. We sought to identify the barriers (recognized by each of these parties) in determining decision making capacity of persons for whom guardianship is being considered. Surveys were conducted with professionals in the legal, medical, and APS arenas, to identify barriers to obtaining capacity determinations. Common themes identified amongst these entities included needing to include functional status in this evaluation, and the need for clear communication between the legal system, medical system, and APS. Unique concerns identified in the survey included APS caseworkers challenges in finding physicians willing to complete capacity evaluations, judges’ concerns about evaluations’ failure to address safety issues and need for immediate action , and physician’s concerns about liability and compromising the physician-patient relationship. Continued collaboration between these systems could overcome these barriers.

